# Exploring Factors Influencing Healthy Aging in Frail Older Adults: A Multidimensional Model Approach

**DOI:** 10.1093/geroni/igaf122.2474

**Published:** 2025-12-31

**Authors:** Rhayun Song, Eunna Oh, Jisu Seo, Yuelin Li

**Affiliations:** Chungnam National University, Daejeon, Republic of Korea; Chungnam National University, Daejeon, Republic of Korea; Chungnam National University, Daejeon, Republic of Korea; University of Pittsburgh, Pittsburgh, Pennsylvania, United States

## Abstract

The concept of healthy aging emphasizes maintaining independence, functional abilities, and overall quality of life, even in the presence of frailty and other age-related challenges. This study aimed to test a hypothesized model based on the multidimensional model of healthy aging and aging-related resilience theory. The model included cognitive and physical function, along with perceived health, as exogenous variables, while resilience, social support, and daily activity acted as mediators in explaining healthy aging among frail older adults. A cross-sectional study was conducted using data from a national survey of 505 frail older adults living in the community in South Korea. Data were collected between October and December 2023 and analyzed using IBM SPSS 26.0 and AMOS 26.0 for structural equation modeling. The model demonstrated a good fit, accounting for 43% of the variance in healthy aging. Resilience, cognitive function, and perceived health had both direct and indirect effects on healthy aging, while daily activity had a direct effect. Social support influenced healthy aging indirectly through daily activity, and physical function had an indirect effect via daily activity. Economic status was also identified as a significant determinant (β = 0.14, p = .016). These findings suggest that cognitive and physical function, mediated by resilience and social support, contribute to healthy aging by promoting daily activity. Health strategies should focus on strengthening resilience and social connectedness to enhance independence. Future research should further explore the impact of social and cultural activities in fostering active aging.

